# Epidemiology, impact and control of bovine cysticercosis in Europe: a systematic review

**DOI:** 10.1186/s13071-016-1362-3

**Published:** 2016-02-10

**Authors:** Minerva Laranjo-González, Brecht Devleesschauwer, Sarah Gabriël, Pierre Dorny, Alberto Allepuz

**Affiliations:** Centre de Recerca en Sanitat Animal (CReSA)-Institut de Recerca i Tecnologia Agroalimentàries (IRTA), Campus UAB, Bellaterra, Barcelona, 08193 Spain; Department of Animal Sciences and Emerging Pathogens Institute, University of Florida, Gainesville, FL USA; Department of Virology, Parasitology and Immunology, Faculty of Veterinary Medicine, Ghent University, Merelbeke, Belgium; Department of Biomedical Sciences, Institute of Tropical Medicine, Antwerp, Belgium; Departament de Sanitat i Anatomia Animals, Universitat Autònoma de Barcelona, Bellaterra, Barcelona, 08193 Spain

**Keywords:** Bovine cysticercosis, *Taenia saginata*, Prevalence, Risk factors, Burden, Cattle, Europe, Systematic review

## Abstract

**Background:**

Bovine cysticercosis in Europe has been known for centuries but the data showing the occurrence of this zoonosis are scarce. The aim of this paper is to review and present the current knowledge on bovine cysticercosis in Europe.

**Methods:**

We conducted a systematic review of studies published between 1990 and November 2014. Qualitative and quantitative data on prevalence, risk factors, burden and interventions were extracted and analysed.

**Results:**

Reports on prevalence were available for 23 European countries, mostly from western and central Europe; for a few of these only data before 1990 were available. Prevalence based on meat inspection was generally low (below 6.2 % in 95 % of the records) and varied between and within countries. Serology and detailed meat inspection provided a higher prevalence range (0.41–14 %). Only few studies analysing risk factors were identified. Reported factors related to access to pastures and risky waters, dairy production and uncontrolled human defecation in the proximity of the farm among others. Only one estimate of the economic impact of the disease could be identified. Recommended interventions were focused on increasing diagnostic tests sensitivity or the application of risk based surveillance strategies.

**Conclusions:**

There is a lack of complete and updated data on most countries, especially in eastern Europe. Further risk factor studies might be needed together with estimates on the burden of the disease in all European countries. Risk-based interventions are being encouraged but current data are limited to guide this approach.

**Electronic supplementary material:**

The online version of this article (doi:10.1186/s13071-016-1362-3) contains supplementary material, which is available to authorized users.

## Background

Bovine cysticercosis is a parasitic infection of cattle caused by the larval stage (cysticercus) of the cestode *Taenia saginata*. Humans are the definitive host and harbour the adult form of the parasite in their intestines. Terminal segments containing eggs are detached from the adult parasite and millions of eggs may be released daily to the environment [1]. Cattle acquire the infection through the ingestion of eggs [2]. The parasite migrates to metabolically active muscles where it develops into cysticerci and humans get infected by consuming raw or undercooked meat containing infective cysticerci.

In cattle, natural infections are normally asymptomatic but they cause financial losses to the cattle industry due to downgrading, condemnation, extra handling, refrigeration and transport of the infected carcasses [3]. The main intervention to control bovine cysticercosis in Europe is meat inspection, followed by condemnation or freezing treatment when necessary, as prescribed by European legislation [4]. However other measures such as thorough cooking of meat and the compliance with the regulations on the treatment and use of wastewater and sludge are determinant to prevent parasite transmission.

The current knowledge of the epidemiological situation of bovine cysticercosis in Europe is mainly based on the detection of cysticerci in the carcasses of bovine animals during meat inspection at the slaughterhouse. In the European Union meat inspection is enforced through Regulation (EC) No 854/2004 which prescribes a visual inspection of specific muscles and incisions in the internal and external masseter muscles (not applicable to animals under six weeks of age) and a lengthwise incision of the heart in cattle of all ages. Carcasses and offal of heavily infected animals (generalised infections) are to be condemned. In the case of lightly infected cattle (localised infections) the affected parts are condemned and the rest of the carcass must undergo a freezing treatment that inactivates the cysticerci [4].

Bovine cysticercosis is distributed worldwide and affects developing and industrialised countries [5]. Official meat inspection reports are considered to be an underestimation of the real prevalence as meat inspection has a low sensitivity for the detection of cysts in muscles [5]. The precision of the visual identification is also questionable, as the cysticerci can be confused with lesions caused by infections with *Sarcocystis* spp. and *Actinobacillus* spp. or with other local alterations [6].

In Europe, the presence of *T. saginata* has been known for centuries, yet data on the occurrence of this zoonosis are scarce, fragmentary and inaccurate with variations regarding the levels of infection in the different countries and regions. The aim of this paper is therefore to review and present the current knowledge of bovine cysticercosis in Europe.

## Methods

### Search strategy

We conducted a systematic review of peer-review papers published from 1990 to November 2014 on the occurrence, risk factors, control measures and burden of bovine cysticercosis in Europe. We followed the PRISMA guidelines for reporting systematic reviews [7] Additional file 1.

In a first step, specific review questions were defined in order to accomplish the scope of the review. The key elements of these review questions were the population (i.e. cattle), exposure (i.e. risk factors or burden), intervention (i.e. control measures) and outcome (i.e. bovine cysticercosis). The search was performed in three international bibliographic databases: PubMed, (on 15 November 2014) and Scopus and Web of Science (on 16 November 2014). The literature search was performed in English using the following set of keywords: ((cattle OR bovine OR beef) AND (cysticerc* OR taeni* OR tapeworm OR tapeworms)) OR “saginata”. For each bibliographic database the search strategy was adapted as follows: in PubMed and Scopus the search was done in “All Fields” and in the Web of Science, it was done in “Topic Field” which includes Title, Abstract and Author Keywords. Retrieved records were exported to an Excel file. Other records reviewed included records obtained through citation searching (publications cited in papers included in the systematic review), the proceedings of the European Network on Taeniosis/Cysticercosis (CYSTINET) meetings, documents published by international institutions (i.e. the European Food Safety Authority publications; Codex Alimentarius guidelines) and unpublished work (i.e. master’s thesis).

**Fig. 1:**
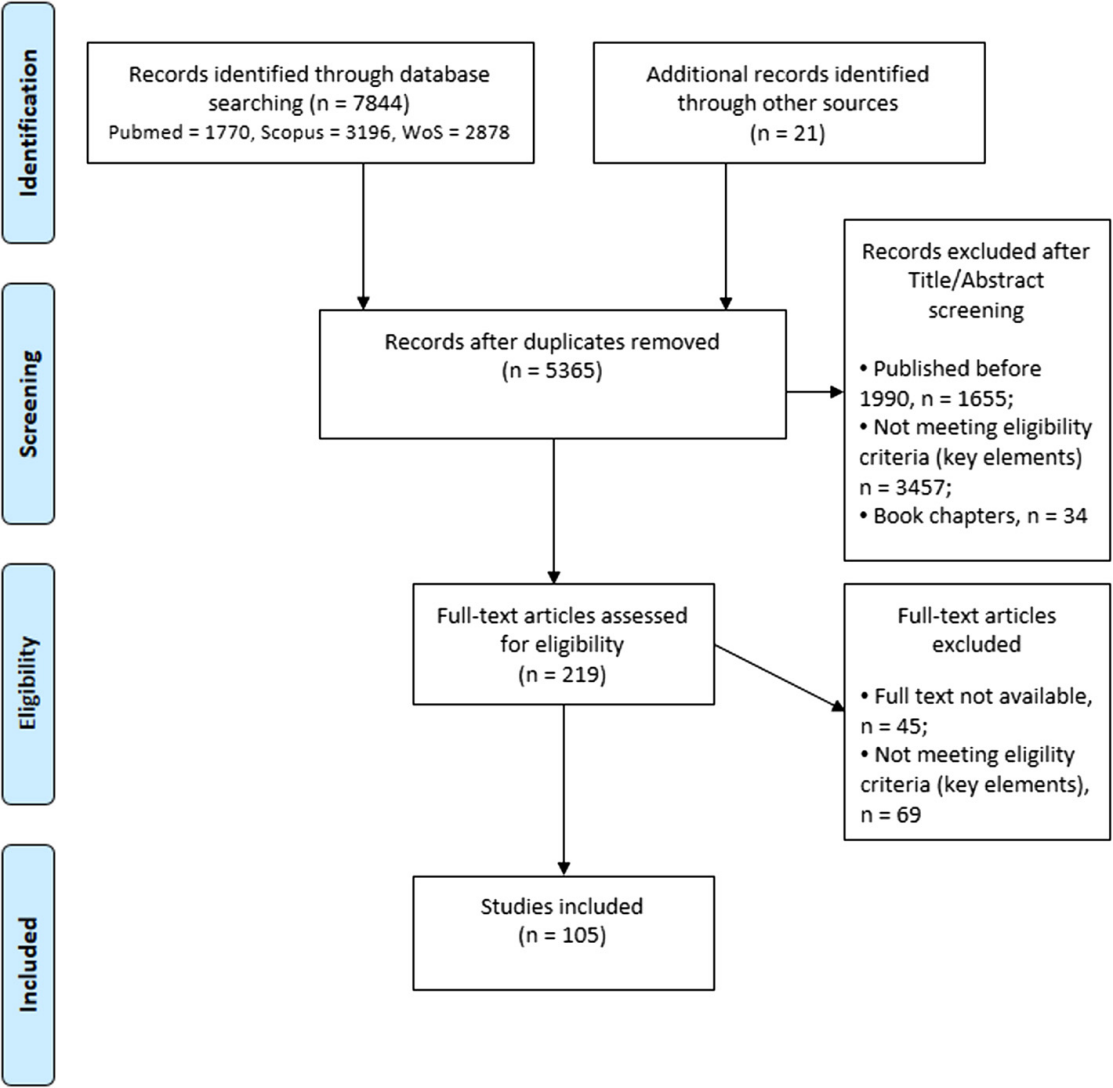
Flow diagram: search strategy steps

In a first screening of all retrieved records, duplicate records were excluded. The titles and abstracts of all unique documents were then screened for relevance to the scope of the review. If the eligibility of the document could not be assessed from the abstract and title only, the full text was screened to exclude or include the document. The exclusion criteria were: (i) publication date before 1990; (ii) wrong agent (other than Cysticercus bovis or *T. saginata*); (iii) wrong host (other than bovine); (iv) providing epidemiological information from outside Europe; (v) providing information different than occurrence, risk factors for *T. saginata* bovine infection and its burden or control measures; and (vi) book chapters. Figure 1 shows the steps applied in the search.

Papers included at this stage were selected for full text revision and assessed for eligibility. Records for which the full text was not available were excluded. Yet, ten of these records provided relevant information in the abstract. This information was included in the review. Records in languages other than Spanish and English were translated with Google Translate (https://www.google.es).

At this step, the screening process was independently assessed by two other reviewers and disagreement about eligibility was discussed among the three reviewers until a consensus was reached. A list with the references included in the review is provided in the Additional file 2.

For each eligible study, quantitative and qualitative data were extracted. Quantitative data regarding prevalence and risk factors were stored in a predefined spread sheet document. Recorded data included information about the country, year of publication, year to which the data belonged, prevalence and 95 % confidence interval (if provided), level of data collection (i.e. national or regional) or measures of association among others. In the case of prevalence, both original and non-original data were collected from the included papers. If the same data were reported in more than one paper these were taken into account only once to avoid duplications. If non-original data were lacking details (e.g. collection date or location), the original source, although not initially included in the review (e.g. study prior to 1990), was consulted unless it was not available.

Data from reports such as the European Food Safety Authority (EFSA) zoonoses reports were used when original source providing the same data (e.g. prevalence for a country in a specific year) had not been included in the review. If the year when the prevalence data were collected was not available the year of the publication of those data was considered instead. Whenever prevalence data corresponded to an interval of years for the purpose of representing it in bar plots only the first year was considered. All the descriptive analyses were performed using R 3.2.0 (https://www.r-project.org/).

Qualitative data on occurrence, risk factors, burden and control measures were extracted and compiled in tables along with the bibliographic reference. These qualitative data were classified according to the type of information given: source of infection in outbreaks, risk factors, protective factors. Identified risk factors were further classified into categories. Relevant information on burden was extracted and summarised in a narrative form. Information on control measures was extracted and grouped into broad categories (i.e. methods to improve sensitivity, measures to destroy eggs, measures to apply to positive farms or preventive measures at farm level among others).

## Results

### Prevalence

We identified bovine cysticercosis prevalence reports for 23 out of the 49 European countries. Most of the data originated from routine inspection and just a few studies reported results based on other diagnostic techniques such as serological tests or detailed meat inspection. A table displaying all the prevalence data identified through the review is provided in Additional file 3.

**Fig. 2:**
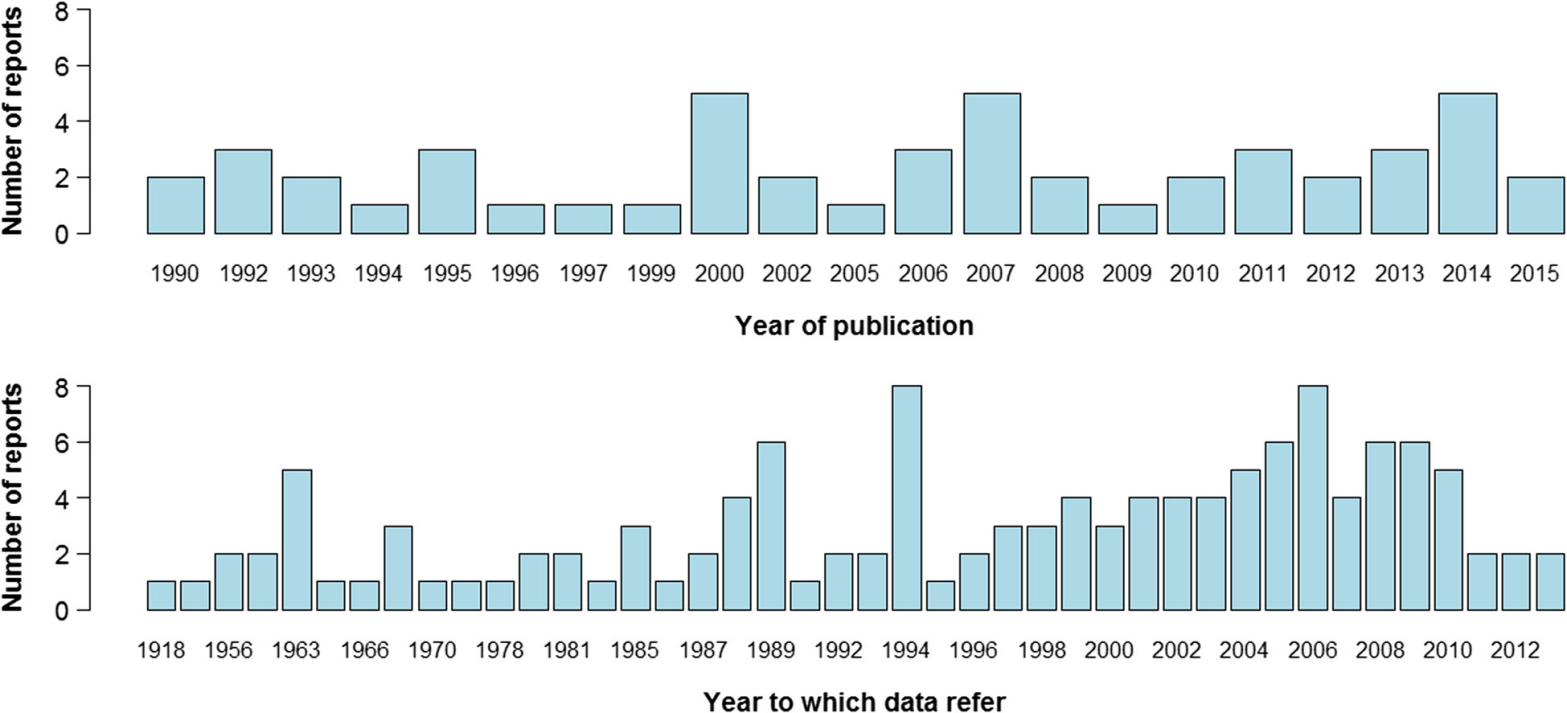
Number of sources reporting prevalence per year of publication and per year of data collection. If data are collected for an interval or years only the value for the first year is presented in the graph

**Fig. 3:**
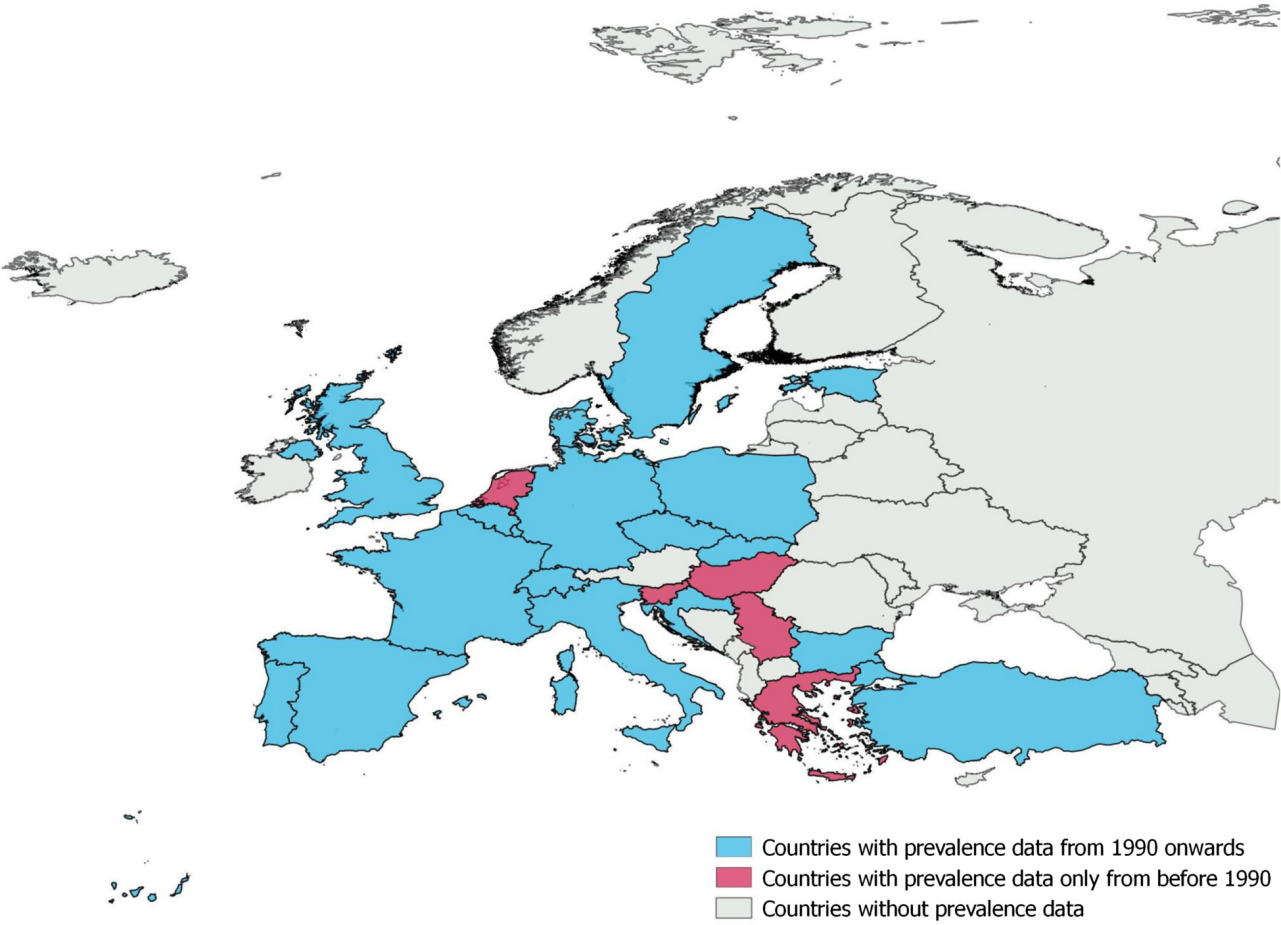
Map of Europe representing availability of prevalence data per country

In total we collected data from 50 different sources reporting bovine cysticercosis prevalence in Europe based on meat inspection. The number of published reports and/or personal communications per year was quite low with no more than three reports in most of the years. Reports showed that bovine cysticercosis has been present in Europe for decades and is still present today (Fig. 2). Most of the data referred to the situation after 1990, since only reports published after 1990 were selected for inclusion. Nevertheless, from the included records we identified data on prevalence from 1918 until 2013 and for some countries such as Greece, Hungary, The Netherlands, Slovenia and Serbia we could only identify reports referring to prevalence prior to 1990 (Fig. 3). The level of prevalence reported by routine meat inspection was generally low across Europe as the prevalence was below 6.2 % in 95 % of the records and below 4.3 % in 90 % of the records.

Few sources provided the age of the animals inspected. Only in a few cases prevalence was given for different groups of age. Results showed higher rates for adult animals than for calves. In a Croatian abattoir during 2005–2010 the prevalence detected in calves (0.014 %) was lower than in steers (0.093 %) and much lower than in cows (0.69 %) [8]. In the UK, during 2008–2011 the prevalence detected in calves and adults was 0.008 and 0.032 %, respectively [9]. These results are in line with the epidemiological situation observed in Belgium where positive cattle are normally adult cattle and calves are generally negative at meat inspection (P. Dorny, personal communication).

Before 1990 the highest rates, based on routine meat inspection, were reported for Turkey, Germany and Poland. In Turkey, the prevalence detected at a regional level ranged from 0.3 to 30 % between 1957 and 1990 [10]. In Eastern Germany and in the province of Olsztyn in Poland prevalences of 3.5–6.8 % and 3.6 %, respectively, were reported during 1974–1989 [11, 12]. After 1990, the highest prevalence levels were reported in one abattoir of Germany (i.e. 6.5 %) in 1992 [13] and in the Autonomous Region of Madeira (i.e. 2.0–5.8 %) during 1993–2005 [14].

**Fig. 4:**
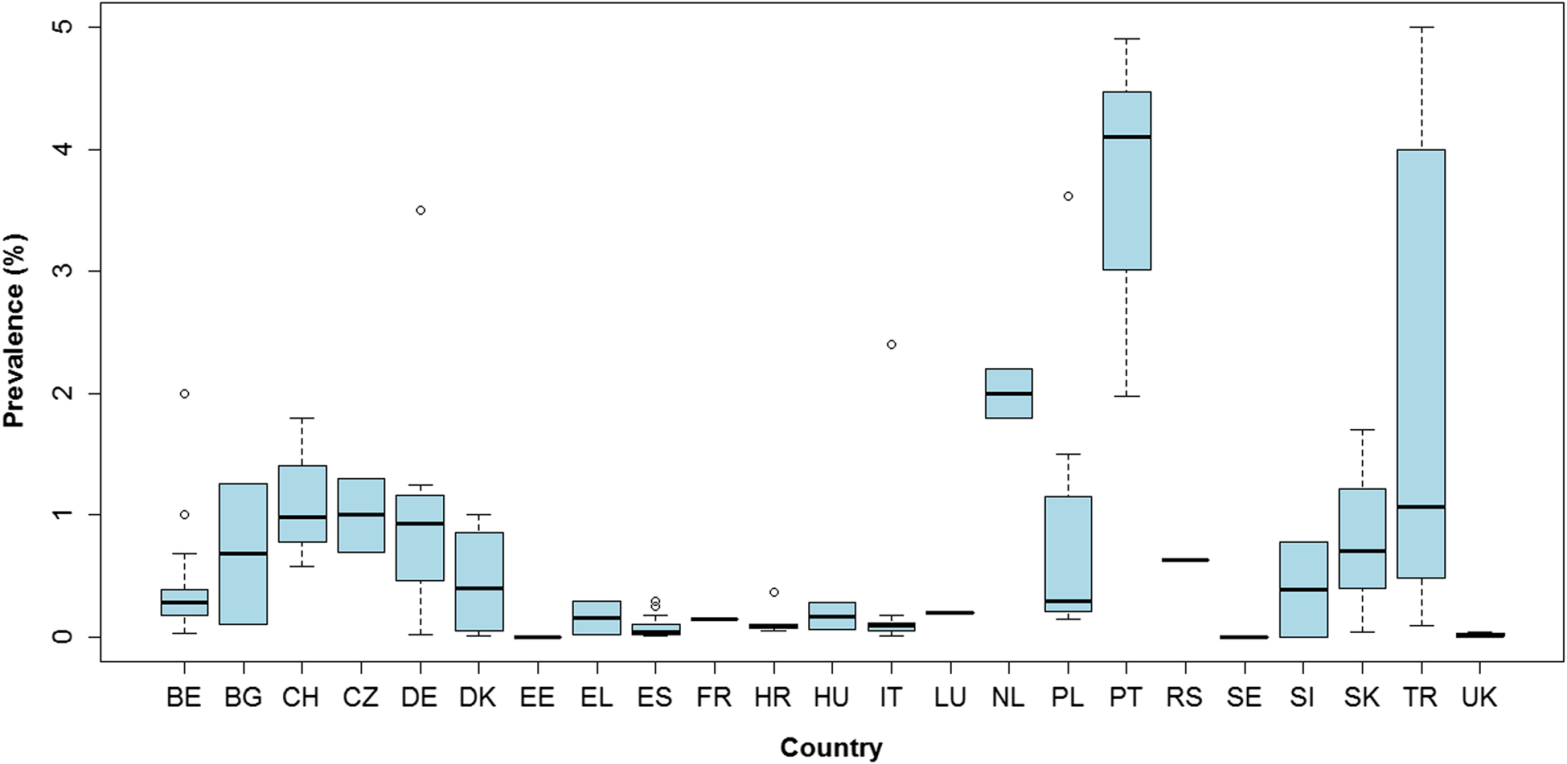
Prevalence levels (%) based on meat inspection reported per country. Prevalences higher than 5 % are not presented in the figure. These data correspond to a few regional records reported in Turkey between 1963 and 1989 (prevalence range 9.7–30 %), one report from the Autonomous Region of Madeira in 2006 (5.8 %) and two reports from Germany (6.5 % in 1992 and 6.8 % between 1974 and 1989). Legend: BE, Belgium; BG, Bulgaria; CH, Switzerland; CZ, Czech Republic; DE, Germany; DK, Denmark; EE, Estonia; ES, Spain; FR, France; UK, United Kingdom; EL, Greece; HR, Croatia; HU, Hungary; IT, Italy; LU, Luxembourg; NL, The Netherlands; PL, Poland; PT, Portugal; SE, Sweden; SK, Slovakia; RS, Serbia; SI, Slovenia; TR, Turkey

The lowest prevalence was identified for Estonia, which reported no positive cases to EFSA for 2006, 2008, 2009 and 2010 [15–17]; followed by Sweden and the UK with a range of 2*10^−4^-1*10^−3^ and 8*10^−3^-4*10^−2^%, respectively [9, 18–20]. In the remaining countries, the prevalence was below 2.0 % with few exceptions (i.e. Italy and The Netherlands). In most of the cases it was below 1.0 %, although the variability between and within countries was very high (Fig. 4).

Few studies reported results based on more sensitive inspection methods such as serology or detailed meat inspection. Studies carried out in Belgium [5] and north-eastern Spain [21] have detected, through antigen ELISA (enzyme-linked immunosorbent assay), a prevalence level 3 to 55 times higher than the prevalence obtained through meat inspection. Also, in Germany, Abuseir et al. [22] performed a regional epidemiological study and detected an antibody titre level of 8.8 %, which is higher than any prevalence level reported through meat inspection in Germany. In Turkey, a prevalence of 14 % resulting from an Indirect fluorescent antibody test (IFA) was reported in the area of Iç Anadolu Bölgesi, City of Konya [10]. Reports based on detailed meat inspection have been reported in Spain, Switzerland and Belgium and show prevalences around 2 to 50 times higher than the prevalence obtained by routine meat inspection [1, 23, 24]. In France, in the Brittany region, in 1973 and 1974 the prevalence by meat inspection was less than 1 % and increased to 9 % when the heart was cut into 2–3 mm-thick slices [25]. Finally, Eichenberger et al. [26] using latent class analysis estimated a prevalence of 16.5 % (95 % CI: 12.5–21.2 %) in dairy cows. This result contrasts with the much lower prevalence estimates resulting from routine meat inspection in Switzerland (Further details presented in Additional file 3).

A recent study performed in Belgium revealed a prevalence of 23 % and 9 % in animals negative to meat inspection by complete cutting of the predilection sites and by antigen ELISA, respectively. Taking into account the sensitivity and specificity of these techniques the authors concluded that around 38.4 % of all carcasses of adult cattle were probably infected with cysticerci (Unpublished observations, Jansen et al., 2015).

### Risk factors

In total, we have found 12 studies that analysed risk factors [5, 8, 23, 27–35]. These studies were conducted in 7 countries, with most studies being conducted in Denmark (3) followed by Belgium, France and Switzerland (2 each) and Croatia, Italy and Spain (1 each).

**Fig. 5:**
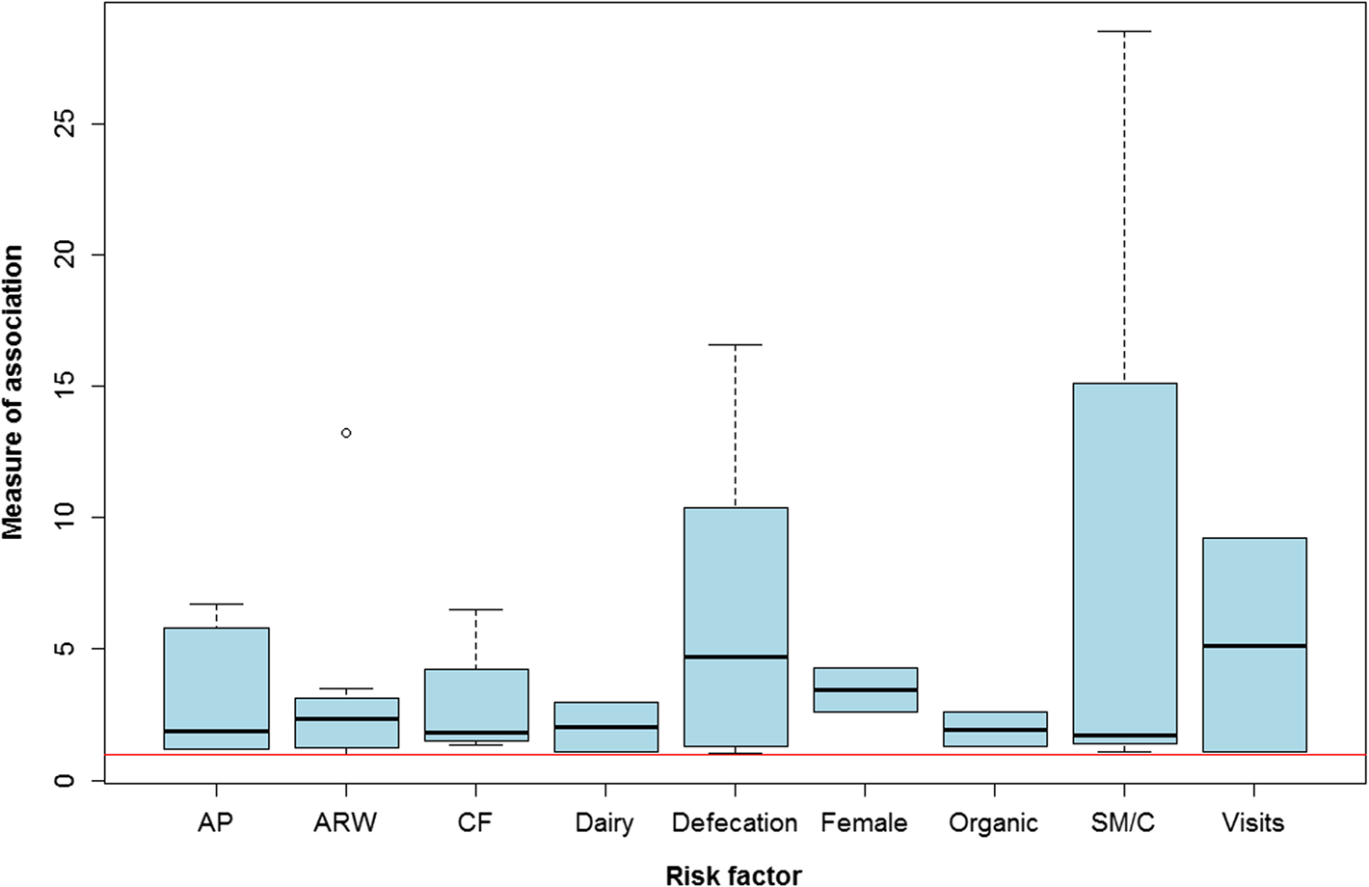
Representation of the degree of association (OR and RR) per each risk factor identified. Only factors associated with a higher risk of infection are represented. The red line sets the point along the Y-axis where the degree of association equals 1. Legend: AP, Access to pastures; ARW, Access to risky water sources; CF, Access to potentially contaminated feed; Dairy, Dairy animals; Female, Being female; Organic, Organic farming; SM/C, Sharing machinery or hiring contractors; Defecation, Proximity to uncontrolled human defecation; Visits, Having visitors on farm

Six of these studies have identified risk factors through the quantification of measures of association (odds ratio or relative risk) between a given factor and the occurrence of cysticercosis. The risk factors at herd level identified in these studies and their level of association with the occurrence of bovine cysticercosis are presented in Fig. 5 (95 % CI represented in most cases). The 95 % CI should be interpreted with caution as small sample sizes might produce wide CI. Further details on the identified risk factors are shown in Table 1.

**Table 1:** Categories of risk factors for bovine cysticercosis represented in Fig. 5

Abbreviation	Risk factor	Subcategories included
AP	Access to pastures	Grazing - organic
		Grazing - conventional (i.e. non-organic)
		Grazing next to streams with single outlets (without direct access to water)
		Grazing next to streams with sewage effluent (without direct access to water)
ARW	Access to risky water sources	Free access to surface water (rivers, lakes, canals)
		Proximity of wastewater effluent (<200 m)
		Access to risky water with sewage treatment plant effluent in proximity^a^
		Access to risky water with no sewage treatment plant effluent in proximity^a^
		Drinking from streams with sewage outlets from single households
		Flooding of pastures
CF	Access to contaminated feed	Feeding of freshly harvested grass on stable to dairy cows
		Purchased roughage
		Sewage sludge spread on own land
Dairy	Dairy animals	Dairy as production type
Female	Being female	Being female
Organic	Organic farming	Organic farming
SM/C	Sharing machinery or hiring contractors	Use of machinery (that had been used for emptying septic tanks) for spreading liquid animal manure
		Sharing machinery or hiring contractors
		Spreading of liquid manure (with machinery that had been used for emptying septic tanks) partially done by contractors
Defecation	Proximity to uncontrolled human defecation	Car park in the proximity of grazing areas
		Leisure activities in the proximity of grazing areas
		Railway line in the proximity of grazing areas
		Distance to camping site ≤ 100 m
Visits	Having visitors on farm	Having visitors on farm

^a^Risky water sources could be streams, lakes or ponds

Eight studies identified other than previously presented risk factors (Fig. 5; Table 1) and for which no association measure was calculated. These risk factors include age and gender [5, 8, 23, 32] . Increasing age and being female have been positively correlated with the occurrence of bovine cysticercosis. At herd level, an increased risk has also been associated with the number of slaughtered animals, the herd size and the location of the herd. Boone et al. [28] observed that the number of slaughtered animals was, among other factors, linked to the occurrence of bovine cysticercosis in a herd. Allepuz et al. [27], Kyvsgaard et al. [36] and Boone et al. [28] found that infected herds had a larger number of animals than uninfected herds. Contradictory results were found in a case–control study conducted by Calvo-Artavia et al. [29] showing that larger herds were less at risk than smaller herds in Denmark. According to the authors, this contradictory result could be due to the fact that in Denmark larger herds are normally kept indoors.

Some studies have investigated the existence of a spatial pattern in the distribution of infected herds. For instance, Allepuz et al. [27] identified two statistically significant clusters in Catalonia, north-eastern Spain. In Belgium, one province was four times less likely to have one infected herd than the three other provinces [28]. In Italy, Cassini et al. [31] identified two significant clusters and Dupuy et al. [33] identified three areas in France with a higher risk of bovine cysticercosis. The reason for disease cluster presence in these areas was attributed to factors such as grazing in mountainous areas with access to risky water sources, movement of infected animals from one infected herd to several herds in the same area or proximity to areas with a high demographic pressure.

In addition to the above mentioned studies, other publications merely hypothesised potential risk factors for bovine cysticercosis without performing any specific study [2, 37, 38]. These factors were related to: (i) access of cattle to contaminated water and/or pastures; (ii) fertilization with potentially contaminated materials; (iii) human defecation in the proximity of grazing areas; (iv) lack of fly and bird control; (v) persistence of eggs in waste water after treatment; (vi) presence of tapeworm carriers in the farm; and (vii) high intensification of agriculture (involving high concentration of cattle and increased irrigation).

Factors linked to a lower probability of cattle becoming infected have also been identified in the literature. Interestingly, Kyvsgaard et al. [35] observed in a case–control study in Denmark a lower risk of infection if sewage sludge was spread on neighbouring land than if no spreading occurred or if the spreading was done on own land. They also identified lower risk if the distance to a sewage treatment plant was ≤ 100 m (in comparison with being at a larger distance) and also being closer than 100 m to a railway track. This last finding is in contrast to the findings of a study conducted in Switzerland where the presence of a railway track along or through farm land was found to increase the risk of infection [34].

### Source of infection

Some studies have performed outbreak investigations to assess potential sources of infection. In Scotland, five outbreaks (1976–1979) were traced back to the application of sludge on grazing fields [39]. However, another survey (1980–1983) investigated affected farms and showed that only in 4.3 % of them sludge had been used, indicating the existence of other routes of infection [20]. A study conducted in Denmark [40] found illegal application of sludge from septic tanks on pasture or crops (in some cases after having been mixed with animal slurry) as the most frequent source of infection. In Spain, by using epidemiological questionnaires and a risk scoring system proposed by EFSA (2004) [24] Allepuz et al. [27] identified that water supply was the most likely source of infection in 23 out of the 55 investigated farms. In Norway, bovine cysticercosis outbreaks have been traced to foreign tourists and seasonal farm workers, and to farm equipment used to handle sewage sludge carrying infected matter [41].

### Burden

According to the literature review, bovine cysticercosis may inflict substantial economic costs to the cattle industry [28, 42, 43] but its impact to public health seems to be less relevant. The clinical importance of *T. saginata* in humans is limited because symptoms are generally mild and it is easily treated [44]. However, severe symptoms can occasionally occur and people carrying a tapeworm can suffer from psychological stress. The main economic losses in the cattle sector are due to extra handling, condemnation, freezing treatment, weight loss after freezing (2–5 %) and loss of value of frozen meat of affected bovines which are reported to be around 30 to 45 % of the value of the carcasses [1]. There is a scarcity of studies quantifying economic burden due to cysticercosis in cattle. In England the costs due to bovine cysticercosis, including condemnation, downgrading, refrigeration, handling, and transport have been estimated at around £100 per carcass or £4.0 million annually [45].

### Interventions

In addition to general control measures described in Reg. (EC) No 854/2004 [4], the systematic review revealed other measures that can be applied. Other measures in place include sewage treatment and the establishment of rules for the agricultural use of sewage and sludge [39] and monitoring of bovine cysticercosis [46]. At farm level, the suggested interventions are to: search for tapeworm carriers among the farm staff [2]; conduct epidemiological studies to find the source of infection in affected farms [8, 31]; and monitor the effectiveness of control measures and provide education and information to farmers. The application of pharmacological treatment to infected herds has also been described as a potential control measure as cattle can be efficiently treated against cysticercosis [47]. However, authors question the feasibility of applying it as the economic cost is high and the degenerated cysticerci can still be present in the carcasses up to two years later. Vaccination has also been proven as an effective tool to protect cattle [48] but vaccines are not commercially available [49] and the cost-benefit is also questioned [50, 51]. Biological control using antagonistic fungi to eliminate *T. saginata* eggs from the environment has been suggested to have potential as a control tool in the future [51].

Due to the very low sensitivity of the current meat inspection procedure the need of applying more sensitive techniques to detect infected cattle has been also highlighted in different studies. Serological tests (based on antibody or antigen detection) provide a better sensitivity. The main downside of antibody detection tests is that they do not distinguish between animals harbouring cysticerci and animals that have been exposed to eggs without establishment of cysticerci (P. Dorny, personal communication). Moreover, low levels of antibodies, antigenic cross-reactivity between parasites and shortage of parasite material as a source of antigen [52] may also occur. Antigen detection tests, detect animals harbouring infective (live) metacestodes [53] but they do not succeed in detecting all light infections, which are the most common type of infection in Europe [5]. Serology is more time consuming than meat inspection but it could be a useful screening test at herd level [47]. AbELISA kits to detect bovine cysticercosis antibodies are currently being commercialised but AgELISA kits are only available for the diagnosis of cysticercosis in humans and pigs and not for cattle. Sensitivity can also be increased, according to previous studies, through increasing the number of incisions in the carcass or in the heart (enhanced meat inspection) [1, 23]. The first would lead to carcass mutilation [21] and to a higher risk of microbiological contamination [21]. The latter, however, would be feasible in the daily practice and useful in low cyst burden areas [23]. A recent study conducted in Belgium showed, however, that performing additional incisions to the heart did not increase the sensitivity of the technique sufficiently to be considered profitable (Jansen et al., 2015, unpublished observations).

Post-mortem laboratory confirmation of *T. saginata* is based on macroscopic, microscopic, histological and molecular assessment of putative lesions. If the lesion is a degenerated cyst or a macroscopically similar lesion caused by other parasites (e.g. *Sarcocystis* spp.) incorrect diagnosis may occur. Different improved post-mortem diagnostic techniques developed for this purpose identified during the review include antigen ELISA in meat juice [54], immunohistochemical methods [6, 55] and biomolecular assays [54, 56].

The interventions to be applied on infected carcasses focus on the destruction of cysts. They include temperature treatment (freezing or cooking of meat) and irradiation. According to an EFSA Scientific Opinion it has been concluded that freezing of cattle carcasses at −10 °C for 10 days kills the cysticerci [57]. It is also generally accepted that cooking meat properly all the way through kills the cysts [57]. Regarding irradiation, the results of a study conducted by Geerts et al. [58] indicated that cysticerci of *T.saginata* lose their infectivity after being irradiated with gamma rays at doses of 0.3, 0.4 and 0.6 kGy.

On the other hand, since classical meat inspection is time-consuming, costly and with low detection sensitivity, several authors have assessed and suggested the application of a risk-based surveillance in order to improve meat inspection sensitivity [25, 30]. This system would consist in implementing a higher priority of surveillance resources in those animals or areas that present higher risk of infection. In this sense, it has been proposed to use more sensitive diagnostic procedures such as the reinforcement of meat inspection (e.g. by using antigen detection serology or increasing the number of incisions in the heart) in high risk areas or animals previously identified as such [33]. For example, in Denmark, Calvo-Artavia et al. [29] proposed including data for the identification of low or high-risk animals in the Food Chain Information document, e.g. gender, age and grazing practices in the case of Denmark, to enable meat inspectors to apply a risk-based inspection. In addition to the use of risk-based surveillance, Dupuy et al. [33] also suggested the application of specific control measures in high-risk areas depending on the risk factors identified (e.g. increasing the control of sewage sludge in areas identified as high-risk areas). Following this approach a Codex Alimentarius document providing guidance on the application of risk-based measures for the control of *T. saginata* in cattle has been recently developed [59].

## Discussion

The high variability in the prevalence levels among and within countries identified through this review could be attributed to different factors. Firstly, real differences might exist due to heterogeneity in the exposure to risk factors among and within countries (e.g. differences in gender, age, herd size, breeding systems, etc.). Secondly, the reported data were collected at different levels (for a whole country, a region, or in one or few abattoirs). For some countries most of the records were recorded at regional level (e.g. Spain or Croatia) whereas in others the prevalence was described mainly at national level (e.g. Belgium or Sweden). In the cases when the level of data collection was not specified, the approach taken was assuming that data belonged to the whole country but this assumption could lead to inaccurate information/interpretation. Thirdly, there were differences in timeframes of data collection. This varied extensively between countries and within a country. Some sources provided a mean prevalence for a long period (e.g. years). In other cases an annual follow-up was given and therefore consecutive annual prevalence data were available (e.g. Belgium). Fourthly, data were extracted from routine official meat inspection reports and from scientific studies. The accuracy of the data derived from a particular scientific study might be higher than the data obtained through official routine meat inspection procedures. Finally, factors influencing the level of detection by routine meat inspection included the training, expertise, motivation of the meat inspector [2], the level of infection (number of cysts), the location of cysts in other muscles than those routinely inspected, the stage of degeneration of the cysts [44], the level of compliance with the officially established meat inspection protocols [4] and also the characteristics of the facilities where the meat inspection is carried out (i.e. speed of slaughter line, lighting, etc.).

Meat inspection sensitivity has been estimated to be between 10 and 30 % [2, 5, 23]; therefore, the data collected underestimate the real prevalence. In order to know the current epidemiological context of bovine cysticercosis the use of more sensitive surveillance strategies is needed and data collection and reporting throughout the years for all of the countries is essential. Monitoring and reporting occurrence of Cysticercus bovis in the European Union is recommended by Directive 2003/99EC (on the monitoring of zoonoses and zoonotic agents) [46], but it is not compulsory and only very few countries annually report their data to the European Commission and European Food Safety Authority.

Only few studies identifying risk factors have been carried out and mostly in western European countries. Since the type of cattle production, farming management and other factors may vary between different parts of Europe, conducting risk factors analysis in Eastern European countries should be encouraged. Also studies based on more sensitive techniques would be needed in order to avoid possible biases due to misclassification of cases [28].

The fact that bovine cysticercosis is present in Europe indicates that the transmission between cattle and humans is taking place and serves also as an indicator of poor hygiene [37, 60]. Human taeniosis is not a notifiable disease and reported prevalences are only indicative [39]. Estimates have indicated that in Europe 11 million people suffer from taeniosis caused by *T. saginata* [61]. Without accurate data on the number of human cases, although the global burden is considered to be low [42, 62], the relevance of bovine cysticercosis as a public health problem is difficult to assess [21] and has not yet been quantified [42]. Few authors have reported estimates of the number of affected humans potentially infected from undetected carcasses during routine meat inspection with variable results. In the UK it was estimated that one human case could originate from between 30–100 undetected bovine cases [9]. In France, however, it was estimated that one undetected carcass could potentially infect between eight and 20 humans [25]. Human taeniosis generally causes mild symptoms (abdominal discomfort, mild diarrhoea, weight loss and anal pruritus) and psychological distress. Only occasionally severe symptoms such as appendicitis occur but no fatalities have been reported. Therefore it is considered that interventions such as meat inspection avert very few Disability-Adjusted Life Years (DALYs) [42]. The only direct cost of human taeniosis is the payment of medical visits, treatment and laboratory tests, which are reported to be very low and reasonable in terms of cost-benefit ratio [42].

There are almost no studies estimating the economic impact of bovine cysticercosis on the meat and cattle industry and in some cases the data are outdated. Earlier research estimated economic losses due to bovine cysticercosis in industrialised countries at 234 US$ for a whole carcass (updated to 1990 US$ prices) [63] but no specific estimates for Europe were provided in that study. In Europe we identified only one estimate on economic impact in England. Therefore, in order to assess the relevance of this animal parasitosis, studies on its economic impact in Europe are needed.

Despite these current control measures, bovine cysticercosis is still present in Europe, which proves that the interventions in place are not sufficient for the successful control of this zoonosis [37]. The current recommendations are to continue performing visual meat inspection until validated serological tests are commercially available for routine practice [37]. In order to better control this parasitosis and also to evaluate the control/prevention tools accurate prevalence data on animals and humans are necessary.

Several authors have suggested the application of risk-based surveillance and control systems to improve the detection sensitivity and to avoid measures that are not proportionate to the level of risk reduction achieved [59]. In order to apply such approaches, classification of areas, herds and animals at low risk, together with the epidemiological data supporting this risk classification are needed. Sources of these data could be records from post-mortem inspection at the slaughterhouses and results from laboratory tests, results from farm investigations, records from human health surveillance and data on human treatments. At present sufficient information to implement such systems are hardly available in Europe, especially in the eastern countries. The quality of data and data reporting of *T. saginata* cysticercosis cases in Europe should be improved. Studies identifying risk factors should be conducted in different countries and for different production systems. This information should allow a better understanding of the epidemiological situation and identification of factors determining level of risk and therefore the implementation of risk-based approaches.

## Conclusions

The available prevalence data for bovine cysticercosis in Europe are scarce and of low quality. This lack of data is especially notable in the eastern countries. There is hardly any knowledge on the economic impact of bovine cysticercosis in Europe. Since current control measures based on meat inspection may not be proportionate to the risk posed according to the epidemiological situation a risk-based surveillance and control approach is currently encouraged. However, the currently available data are limited to guide such an approach.

## Additional files

Additional file 1:
**PRISMA 2009 Checklist.** (DOC 65 kb) Additional file 2:
**List of references for the records included in the review.** (XLSX 20 kb) Additional file 3:
**Prevalence data extracted from the included records.** (DOCX 98 kb)

## Abbreviations

CI: Confidence interval
CYSTINET: European Network on Taeniosis/Cysticercosis
EFSA: European food safety authority
ELISA: Enzyme-linked immunosorbent assay
DALYs: Disability-adjusted life years
OR: Odds ratio
RR: Relative risk
