# Supplementary material for: Epidemiology, impact and control of bovine cysticercosis in Europe: a systematic review
Source: Parasit Vectors. 2016 Feb 10;9:81. doi: 10.1186/s13071-016-1362-3 (PMC4748494; doi:10.1186/s13071-016-1362-3)
Supplement: Additional file 3: — Prevalence data extracted from the included records. (DOCX 98 kb) [file 13071_2016_1362_MOESM3_ESM.docx]

**Table 1**. Prevalence data based on meat inspection

| **Country** | **Prevalence (%)** | **Reference** | **Timeframe** | **Level of data collection^1^** |
| --- | --- | --- | --- | --- |
| Belgium | 0.03–0.30^2^ | [[1](#_ENREF_1)] | 1969-1989 | National level |
| Belgium | 1.00-2.00 | [[1](#_ENREF_1)] | 1969-1989 | Regional level (1 abattoir in Melle) |
| Belgium | 0.26 | [[2](#_ENREF_2)] | 1997-1998 | National level |
| Belgium | 0.22 | [[3](#_ENREF_3)] | 2001 | National level |
| Belgium | 0.44-0.68 | [[3](#_ENREF_3), [4](#_ENREF_4)] | 2003 | National level |
| Belgium | 0.34 | [[5](#_ENREF_5)] | 2004 | National level |
| Belgium | 0.29 | [[6](#_ENREF_6)] | 2005 | National level |
| Belgium | 0.20 | [[7](#_ENREF_7)] | 2006 | National level |
| Belgium | 0.30 | [[8](#_ENREF_8)] | 2008 | National level |
| Belgium | 0.16 | [[9](#_ENREF_9)] | 2011 | National level |
| Belgium | 0.15 | [[9](#_ENREF_9)] | 2012 | National level |
| Belgium | 0.12 | [[9](#_ENREF_9)] | 2013 | National level |
| Bulgaria | 0.10 | [[10](#_ENREF_10)] | 1989 | National level |
| Bulgaria | 1.26 | [[10](#_ENREF_10)] | 1994 | National level |
| Croatia | 0.37 | [[11](#_ENREF_11)] | 2005 | Regional level (1 abattoir) |
| Croatia | 0.049 | [[11](#_ENREF_11)] | 2006 | Regional level (1 abattoir) |
| Croatia | 0.10 | [[11](#_ENREF_11)] | 2007 | Regional level (1 abattoir) |
| Croatia | 0.10 | [[11](#_ENREF_11)] | 2008 | Regional level (1 abattoir) |
| Croatia | 0.07 | [[11](#_ENREF_11)] | 2009 | Regional level (1 abattoir) |
| Croatia | 0.067 | [[11](#_ENREF_11)] | 2010 | Regional level (1 abattoir) |
| Czech Republic | 0.70 | [[12](#_ENREF_12)] | 1989 | National level |
| Czech Republic | 1.30 | [[12](#_ENREF_12)] | 1998 | National level |
| Denmark | 1.00 | [[12](#_ENREF_12)] | 1918-1982 | National level |
| Denmark | 0.009 | [13] | 2004-2011 | National level |
| Denmark | 0.10 | [14] | 1988** | National level |
| Denmark | 0.70 | [14] | 1988* | Regional level (Southern part of Jutland) |
| Estonia | 0.00 | [[7](#_ENREF_7)] | 2006 | National level |
| Estonia | 0.00 | [[8](#_ENREF_8)] | 2008 | National level |
| Estonia | 0.00 | [15] | 2009 | National level |
| Estonia | 0.00 | [16] | 2010 | National level |
| France | 0.14 | [17] | 2010 | National level (181 abattoirs) |
| Germany | 3.50–6.80 | [18] | 1974-1989 | Regional level (Former German Democratic Republic) |
| Germany | 0.44–1.24 | [18] | 1970-1990 | Regional level (Former West Germany) |
| Germany | 0.48 and 1.08  (2 abbatoirs, respectively) | [19] | 2004 | Regional level (Northern Germany, 2 abattoirs) |
| Germany | 0.02 | [20] | 2007 | National level |
| Germany | 6.49^3^ | [21] | 1992* | Regional level (1 abattoir) |
| Germany | 0.93^4^ | [21] | 1992* | Regional level (1 abattoir) |
| Greece | 0.29 | [[12](#_ENREF_12)] | Before 1969 | National level |
| Greece | 0.02 | [[12](#_ENREF_12)] | After 1978 | National level |
| Hungary | 0.28 | [[12](#_ENREF_12)] | 1956 | National level |
| Hungary | 0.06 | [[12](#_ENREF_12)] | 1983 | National level |
| Italy | 0.10 | [[12](#_ENREF_12)] | 1946–1955 | Regional level (Region of Torino) |
| Italy | 0.09 | [[12](#_ENREF_12)] | 1956–1965 | Regional level (Region of Torino) |
| Italy | 0.12 | [[12](#_ENREF_12)] | 1966–1975 | Regional level (Region of Torino) |
| Italy | 0.02–2.40 | [22] | 1999** | National level |
| Italy | 0.06 (CI:95% = 0.05-0.08) | [23] | 2006-2010 | Regional level (Region of Veneto) |
| Italy | 0.065–0.179 | [24] | 1993 - 2003 | Regional level (Province of Ravenna, 1 abattoir) |
| Italy | 0.039–0.097 | [[14](#_ENREF_14)] | 1998-2003 | Regional level (Province of Ravenna, 1 abattoir) |
| Italy | 0.01 | [20] | 2007 | National level |
| Luxembourg | 0.20 | [[7](#_ENREF_7)] | 2006 | National level |
| The Netherlands | 1.80-2.20 | [22] | 1985* | National level |
| Poland | 0.18 | [25] | 1986-1994 | Regional level (Katowice district) |
| Poland | 0.31-1.50 | [26] | 1994 | National level |
| Poland | 0.25 | [27] | 1997 | National level |
| Poland | 0.24 | [22] | 1999** | National level |
| Poland | 0.29-1.49 (mean=0.44) | [28] | 2005–2008 | Regional level (Province of Lublin) |
| Poland | 0.81 | [29] | 1985 | Regional level (Province of Katowice) |
| Poland | 3.62 | [30] | 1980-1991 | Regional level (Province of Olsztyn) |
| Poland | 0.14 | [31] | 1987-1992 | Regional level (Region of Kielce) |
| Poland | 0.18 | [32] | 2000 | Regional level (Various provincial veterinary inspectorates) |
| Portugal | 3.01 | Unpublished results (Da Fonseca and Spínola, 2000) | 1993 | Regional level (Autonomous Region of Madeira) |
| Portugal | 1.98 | Unpublished results (Da Fonseca and Spínola, 2000) | 1994 | Regional level (Autonomous Region of Madeira) |
| Portugal | 2.26 | Unpublished results (Da Fonseca and Spínola, 2000) | 1995 | Regional level (Autonomous Region of Madeira) |
| Portugal | 2.84 | Unpublished results (Da Fonseca and Spínola, 2000) | 1996 | Regional level (Autonomous Region of Madeira) |
| Portugal | 4.91 | Unpublished results (Da Fonseca and Spínola, 2000) | 1997 | Regional level (Autonomous Region of Madeira) |
| Portugal | 4.76 | Unpublished results (Da Fonseca and Spínola, 2000) | 1998 | Regional level (Autonomous Region of Madeira) |
| Portugal | 3.11 | [33] | 1999 | Regional level (Autonomous Region of Madeira) |
| Portugal | 4.47 | [33] | 2000 | Regional level (Autonomous Region of Madeira) |
| Portugal | 3.74 | [33] | 2001 | Regional level (Autonomous Region of Madeira) |
| Portugal | 4.51 | [33] | 2002 | Regional level (Autonomous Region of Madeira) |
| Portugal | 4.10 | [33] | 2003 | Regional level (Autonomous Region of Madeira) |
| Portugal | 4.12 | [33] | 2004 | Regional level (Autonomous Region of Madeira) |
| Portugal | 4.30 | [33] | 2005 | Regional level (Autonomous Region of Madeira) |
| Portugal | 5.83 | [33] | 2006 | Regional level (Autonomous Region of Madeira) |
| Serbia | 0.63 | [34] | 1988-1989 | Regional level (Pozarevac, 1 abattoir) |
| Slovakia | 0.93 | [35] | 1988-1989 | National level |
| Slovakia | 0.50 | [35] | 1994 | National level |
| Slovakia | 0.30 | [35] | 1989 | Regional level (Western Slovakia) |
| Slovakia | 0.04 | [35] | 1994 | Regional level (Western Slovakia) |
| Slovakia | 1.70 | [35] | 1989 | Regional level (Central Slovakia) |
| Slovakia | 0.79 | [35] | 1994 | Regional level (Central Slovakia) |
| Slovakia | 1.50 | [35] | 1989 | Regional level (Eastern Slovakia) |
| Slovakia | 0.63 | [35] | 1994 | Regional level (Eastern Slovakia) |
| Slovenia | 0.0-0.78 | [36] | 1965-1991 | Regional level (Murska Sobota, 1 abattoir) |
| Spain | 0.007–0.10 | Personal communication (Garate, 1999) | 1999** | Regional level (Castilla y León) |
| Spain | 0.05 | [37] | 2000 | Regional level (Province of Gipuzkoa) |
| Spain | 0.09 | [37] | 2001 | Regional level (Province of Gipuzkoa) |
| Spain | 0.18 | [37] | 2002 | Regional level (Province of Gipuzkoa) |
| Spain | 0.29 | [37] | 2003 | Regional level (Province of Gipuzkoa) |
| Spain | 0.25 | [37] | 2004 | Regional level (Province of Gipuzkoa) |
| Spain | 0.10 | [37] | 2005 | Regional level (Province of Gipuzkoa) |
| Spain | 0.015 | [38] | 2005 | Regional level (Catalonia) |
| Spain | 0.017 | [38] | 2006 | Regional level (Catalonia) |
| Spain | 0.022 | [38] | 2007 | Regional level (Catalonia) |
| Spain | 0.021 | [38] | 2008 | Regional level (Catalonia) |
| Spain | 0.016 | Personal communication (Allepuz, 2014) | 2009 | Regional level (Catalonia) |
| Spain | 0.010 | Personal communication (Allepuz, 2014) | 2010 | Regional level (Catalonia) |
| Sweden | 0.001  (*Cysticercus* spp.) | [15] | 200 | National level |
| Sweden | 0.0007 | [16] | 2010 | National level |
| Sweden | 0.0002 | [39] | 2011 | National level |
| Sweden | 0.0002 | [39] | 2012 | National level |
| Sweden | 0.0002 | [[9](#_ENREF_9)] | 2013 | National level |
| Switzerland | 1.00 | [40] | 1996* | National level |
| Switzerland | 0.58 | [41] | 2002-2005 | Regional level (6 abattoirs) |
| Switzerland | 0.97 | [42] | 2002-2005 | Regional level (6 abattoirs) |
| Switzerland | 1.80 | [42] | 2008-2009 | National level |
| Turkey | 0.70 | [43] | 1989* | Regional level (Area of Marmara Bölgesi, City of Bursa) |
| Turkey | 1.00-10.00 | [43] | 1963*, 1989* | Regional level (Area of Marmara Bölgesi, City of Istanbul) |
| Turkey | 0.50-4.00 | [43] | 1963*, 1989*, 1981* | Regional level (Area of Ege Bölgesi, City of Izmir) |
| Turkey | 1.13 | [43] | 1994* | Regional level (Area of Ege Bölgesi, City of Manisa) |
| Turkey | 0.46 | [44] | 2009-2011 | Regional level (Area of Ege Bölgesi, City of Afyonkarahisar) |
| Turkey | 0.09 | [44] | 2009-2011 | Regional level (Area of Akdeniz Bölgesi, City of Burdur) |
| Turkey | 0.30-9.70 | [43] | 1963*, 1998* | Regional level (Area of Iç Anadolu Bölgesi, City of Ankara) |
| Turkey | 4.70 | [43] | 1985-1988 | Regional level (Infected cattle of City of Sivas, Tokat and Erzincan) |
| Turkey | 2.60 | [43] | 1963* | Regional level (Area of Iç Anadolu Bölgesi, City of Konya) |
| Turkey | 2.10 | [43] | 1990* | Regional level (Area of Karadeniz Bölgesi, City of Samsun) |
| Turkey | 10.00-20.00 | [43] | 1963*, 1989* | Regional level (Area of Doğu Anadolu Bölgesi, City of Erzurum) |
| Turkey | 4.00 | [43] | 1957* | Regional level (Area of Doğu Anadolu Bölgesi, City of Kars) |
| Turkey | 5.00 | [43] | 1957* | Regional level (Area of Doğu Anadolu Bölgesi, City of Ağn) |
| Turkey | 0.55-4.30^5^ | [43] | 1987*,  1988-1993 | Regional level (Area of Doğu Anadolu Bölgesi, City of Elaziğ) |
| Turkey | 0.34 | [43] | 1981-1990 | Regional level (Area of Doğu Anadolu Bölgesi, City of Van) |
| Turkey | 25.00-30.00 | [43] | 1981* | Regional level (Area of Güneydoğu Anadolu, City of Sanliurfa) |
| United Kingdom | 0.04 | [45] | 1980-1990 | Regional level (England and Wales) |
| United Kingdom | 0.00 | [20] | 2006 | National level |
| United Kingdom | 0.011 (CI:95% = 0.011-0.012) | [46] | 2001-2011 | Regional level (Northern Ireland, 10 abattoirs) |
| United Kingdom | 0.008 and 0.032 (calves and adults respectively) | [47] | 2008-2011 | National level |

*year of publication of data (year of data collection unspecified)

**year of personal communication

^1^ When data collection level has not been specified it has been assumed it corresponds to national level.

^2^ Prevalence intervals correspond to ranges of observations and only in few cases the confidence interval is given (specified in the table).

^3,4^ Prevalence extracted from abstract as full text was not available; country of data collection was not specified. It has been assumed it belongs to Germany (author based in Germany and article published in a German speaking journal).

^5^ Results include inspection of buffalo

**Table 2**. Prevalence based on diagnostic methods different than routine meat inspection

| **Country** | **Prevalence (%)** | **95% CI** | **Reference** | **Timeframe** | **Diagnostic method** | **Level of data collection** |
| --- | --- | --- | --- | --- | --- | --- |
| Belgium | 3.09 (range: 0.9-5.0) | - | [2] | 1997-1998 | Ag-ELISA | National level (20 abattoirs) |
| Germany | 8.83 (range: 1.61-33.3) | - | [48] | 2007-2008 | Ab-ELISA | Regional level (Federal state of Lower Saxony in Germany) |
| Spain | 1.11 | 0.76–1.75 | [49] | 2009-2010 | Ag-ELISA | Regional level (Catalonia) |
| Turkey | 14.00 | - | [43] | 1999* | Indirect fluorescent antibody (IFA) test | Regional level (Area of Iç Anadolu Bölgesi, City of Konya) |
| Switzerland | 4.50 | - | [42] | 2008-2009 | Detailed meat inspection (additional heart incisions) | National level |
| Spain | Mean = 0.54 (range: 0.41-0.75) | - | [50] | 1992-1998 | Detailed meat inspection | Regional (Northern spain) |
| Belgium | 9.50 | - | [51] | 1990* | Detailed meat inspection | National level |
| Switzerland | 16.50 | 12.50–21.20 | [52] | - | Obligatory meat inspection and 4 serological tests  (Prevalence estimated by latent class analysis) | Estimated prevalence for abattoirs in Switzerland |

*year of publication of data (year of data unspecified)

**References**

1. Geerts S, Brandt J, Kumar V, De Deken R. Immunodiagnosis of *Taenia saginata* cysticercosis. Verh K Acad Geneeskd Belg. 1992; 54(4):329-343; discussion 343-326.

2. Dorny P, Vercammen F, Brandt J, Vansteenkiste W, Berkvens D, Geerts S. Sero-epidemiological study of T*aenia saginata* cysticercosis in Belgian cattle. Veterinary parasitology. 2000; 88(1-2):43-49.

3. Boone I, Thys E, Marcotty T, de Borchgrave J, Ducheyne E, Dorny P. Distribution and risk factors of bovine cysticercosis in Belgian dairy and mixed herds. Preventive veterinary medicine. 2007; 82(1-2):1-11.

4. Geysen D, Kanobana K, Victor B, Rodriguez-Hidalgo R, De Borchgrave J, Brandt J, Dorny P. Validation of meat inspection results for *Taenia saginata* cysticercosis by PCR-restriction fragment length polymorphism. J Food Prot. 2007; 70(1):236-240.

5. The Community Summary Report on Trends and Sources of Zoonoses, Zoonotic Agents and Antimicrobial Resistance in the European Union in 2004. EFSA Journal. 2005; 310.

6. The Community Summary Report on Trends and Sources of Zoonoses, Zoonotic Agents, Antimicrobial Resistance and Foodborne Outbreaks in the European Union in 2005. EFSA Journal. 2006; 94.

7. The Community Summary Report on Trends and Sources of Zoonoses, Zoonotic Agents, Antimicrobial Resistance and Foodborne Outbreaks in the European Union in 2006. EFSA Journal. 2007; 130.

8. The Community Summary Report on Trends and Sources of Zoonoses, Zoonotic Agents and Food-borne Outbreaks in the European Union in 2008. EFSA Journal. 2010; 8(1):1496.

9. The European Union Summary Report on Trends and Sources of Zoonoses, Zoonotic Agents and Food-borne Outbreaks in 2013. EFSA Journal. 2015; 13(1):3991.

10. Kanev I, Petrov P, Komandarev S, Boeva V, Kurdova R, Tanchev T, et al. Basic helminthological issues in Eastern Europe after the democratic changes. Helminthologia. 1995; 32(3):117-120.

11. Zdolec N, Vujevic I, Dobranic V, Juras M, Grgurevic N, Ardalic D, Njari B. Prevalence of *Cysticercus bovis* in slaughtered cattle determined by traditional meat inspection in Croatian abattoir from 2005 to 2010. Helminthologia. 2012; 49(4):229-232.

12. Cabaret J, Geerts S, Madeline M, Ballandonne C, Barbier D. The use of urban sewage sludge on pastures: the cysticercosis threat. Vet Res. 2002; 33(5):575-597.

13. Calvo-Artavia FF, Nielsen LR, Dahl J, Clausen DM, Alban L. Occurrence and factors associated with bovine cysticercosis recorded in cattle at meat inspection in Denmark in 2004-2011. Preventive veterinary medicine. 2013; 110(2):177-182.

14. Ilsoe B, Kyvsgaard NC, Nansen P, Henriksen SA. Bovine cysticercosis in Denmark. A study of possible causes of infection in farms with heavily infected animals. Acta Vet Scand. 1990; 31(2):159-168.

15. The European Union Summary Report on Trends and Sources of Zoonoses, Zoonotic Agents and Food-borne Outbreaks in 2009. EFSA Journal. 2011; 9(3):2090.

16. The European Union Summary Report on Trends and Sources of Zoonoses, Zoonotic Agents and Food-borne Outbreaks in 2010. EFSA Journal. 2012; 10(3):2597.

17. Dupuy C, Morlot C, Demont P, Callait-Cardinal MP, Ducrot C, Calavas D, Gay E. Spatial analysis of bovine cysticercosis in France in 2010. Food Control. 2015; 47:348-352.

18. Mobius G. Epidemiologic studies of *C. bovis* and *T. saginata* infections in eastern and western Germany. DTW Deutsche tierarztliche Wochenschrift. 1993; 100(3):110-114.

19. Abuseir S, Epe C, Schnieder T, Klein G, Kuhne M. Visual diagnosis of *Taenia saginata* cysticercosis during meat inspection: is it unequivocal? Parasitol Res. 2006; 99(4):405-409.

20. Development of harmonised schemes for the monitoring and reporting of Cysticercus in animals and foodstuffs in the European Union. Scientific report submitted to EFSA 2010.

21. Ring C. Environmental and food hygiene aspects of cestode infections of humans. DTW Deutsche tierarztliche Wochenschrift. 1992; 99(7):295-297.

22. [SCVMPH](http://www.abbreviations.com/term/220956). Opinion of the Scientific Committee on Veterinary Measures relating to Public Health on The control of taeniosis/cysticercosis in man and animals (adopted on 27-28 September 2000). European Commission; 2000.

23. Cassini R, Mulatti P, Zanardello C, Simonato G, Signorini M, Cazzin S, et al. Retrospective and spatial analysis tools for integrated surveillance of cystic echinococcosis and bovine cysticercosis in hypo-endemic areas. Geospat Health. 2014; 8(2):509-515.

24. Padovani A PA, Trevisani M and Bettini G. Occurrence of Taenia saginata cysticercosis in slaughtered cattle in the North of Italy: results of a ten-year monitoring program. In: Food safety assurance and veterinary public health. Edited by Smulders F, vol. 4: Wageningen Academic Publishers; 2006: 329–331.

25. Derylo A, Szilman P. Occurrence of human taeniasis and cysticercosis in pigs and cattle in the Katowice district. Wiadomosci parazytologiczne. 1995; 41(4):443-454.

26. Lis H. Evaluation of veterinary inspection of slaughtered cattle in Poland. Medycyna Weterynaryjna. 1997; 53(3):155-158.

27. Lis H. An evaluation of veterinary inspection of slaughtered animals and meat in Poland between 1987-1997. Medycyna Weterynaryjna. 1999; 55(4):243-246.

28. Kozlowska-Loj J. Prevalence of cysticercosis in cattle and pigs in the Lublin province in the years 2005-2008. Wiadomosci parazytologiczne. 2011; 57(3):193-194.

29. Michalski MM. Economic losses caused by parasite invasions in animals and methods of their evaluation. Medycyna Weterynaryjna. 2007; 63(6):643-647.

30. Uradzinski J, Radkowski M. The occurrence of parasitic invasions in slaughter animals in the Olsztyn province in years 1980-1991. Medycyna Weterynaryjna. 1992; 48(12):564-566.

31. Konopka B. The incidence of pathogenic parasites in slaughter animals in the Kielce region in 1987-1992. Medycyna Weterynaryjna. 1993; 49(8):373-374.

32. Lis H. Results of veterinary inspection of slaughtered animals and meat in Poland in 2000. Medycyna Weterynaryjna. 2002; 58(4):267-269.

33. Afonso M. Prevalência de *Taenia saginata/Cysticercus bovis* na Região Autónoma da Madeira. Universidade Técnica de Lisboa, Faculdade de Medicina Veterinária; 2008.

34. Aleksic N, Miloradovic S. Cysticercosis of cattle slaughtered in the abattoir of the meat industry "Pozarevac" in Pozarevac. Veterinarski Glasnik. 1994; 48(9):751-756.

35. Stefancikova A, Dubinsky P. Status and prognosis of the incidence of helminthic zoonoses in Slovakia. Helminthologia. 1995; 32(4):247-250.

36. Zivkovic J, Velimirovic D, Dzaja P, Grabarevic Z. Prevalence of *Cysticercus bovis s inermis* measles with particular reference to histopathological changes in meat. Archiv Fur Lebensmittelhygiene. 1996; 47(3):66-68.

37. Artieda J, Álvarez L, Abadia L, Cortes-Alonso A, Echeverria MJ: Investigación epidemiológica de 4-5 *Taenia saginata* y cysticercosis bovis en Gipuzkoa. Año 2005. Boletín Epidemiológico del País Vasco Salud Pública. 2006; Número 20(1er semestre):4-5.

38. Allepuz A, Napp S, Picado A, Alba A, Panades J, Domingo M, Casal J. Descriptive and spatial epidemiology of bovine cysticercosis in North-Eastern Spain (Catalonia). Veterinary parasitology. 2009; 159(1):43-48.

39. The European Union Summary Report on Trends and Sources of Zoonoses, Zoonotic Agents and Food-borne Outbreaks in 2012. EFSA Journal. 2014; 12(2):3547.

40. Van der Logt PB, Gottstein B. Unidentified parasitic cysts in cattle. Vet Rec. 2000; 146(21):610-612.

41. Flutsch F, Heinzmann D, Mathis A, Hertzberg H, Stephan R, Deplazes P. Case-control study to identify risk factors for bovine cysticercosis on farms in Switzerland. Parasitology. 2008; 135(5):641-646.

42. Eichenberger RM, Stephan R, Deplazes P. Increased sensitivity for the diagnosis of T*aenia saginata* cysticercus infection by additional heart examination compared to the EU-approved routine meat inspection. Food Control. 2011; 22(6):989-992.

43. Kus FS, Sevimli FK, Miman O. *Cysticercus bovis* in Turkey and its importance from the public health aspect. Turkiye parazitolojii dergisi / Turkiye Parazitoloji Dernegi = Acta parasitologica Turcica / Turkish Society for Parasitology. 2014; 38(1):41-47.

44. Kus FS, Sevimli FK, Miman O. *Cysticercus bovis* in slaughtered cattle in the Afyonkarahisar and Burdur provinces and its importance from the point of view of public health. Turkiye parazitolojii dergisi / Turkiye Parazitoloji Dernegi = Acta parasitologica Turcica / Turkish Society for Parasitology. 2013; 37(4):262-268.

45. Bruce AM, Pike EB, Fisher WJ. A review of treatment process options to meet the EC sludge directive. Journal of the Institution of Water and Environmental Management. 1990; 4(1):1-13.

46. McBrien J, Courcier EA. Detection of *Cysticercus bovis* in abattoirs in Northern Ireland between 2001 and 2011. Vet Rec. 2013; 173(12):296.

47. Hill AA, Horigan V, Clarke KA, Dewe TCM, Staerk KDC, O'Brien S, Buncic S. A qualitative risk assessment for visual-only post-mortem meat inspection of cattle, sheep, goats and farmed/wild deer. Food Control. 2014; 38:96-103.

48. Abuseir S, Nagel-Kohl U, Probst D, Kuhne M, Epe C, Doherr MG, Schnieder T. Seroprevalence of *Taenia saginata* cysticercosis in the federal state of Lower Saxony in Germany. Berliner und Munchener tierarztliche Wochenschrift. 2010; 123(9-10):392-396.

49. Allepuz A, Gabriel S, Dorny P, Napp S, Jansen F, Vilar MJ, et al. Comparison of bovine cysticercosis prevalence detected by antigen ELISA and visual inspection in the North East of Spain. Res Vet Sci. 2012; 92(3):393-395.

50. Opinion of the Scientific Panel on Biological Hazards on “Risk assessment of a revised inspection of slaughter animals in areas with low prevalence of *Cysticercus*". The EFSA Journal. 2004; 176.

51. Geerts S. *Taenia saginata*: an eternal problem? Verh K Acad Geneeskd Belg. 1990; 52(6):537-563; discussion 563-534.

52. Eichenberger RM, Lewis F, Gabriel S, Dorny P, Torgerson PR, Deplazes P. Multi-test analysis and model-based estimation of the prevalence of *Taenia saginata* cysticercus infection in naturally infected dairy cows in the absence of a 'gold standard' reference test. Int J Parasitol. 2013; 43(10):853-859.
